# The (CF_3_C(O)NH)(C_6_H_5_CH_2_NH)_2_P(O) phosphoric triamide as a novel carrier with excellent efficiency for Cu(ii) in a liquid membrane transport system†

**DOI:** 10.1039/c8ra09118h

**Published:** 2019-03-19

**Authors:** Setareh Akbari, Razieh Sanavi Khoshnood, Fatemeh Karimi Ahmadabad, Mehrdad Pourayoubi, Michal Dušek, Ekaterina S. Shchegravina

**Affiliations:** Department of Chemistry, Mashhad Branch, Islamic Azad University Mashhad Iran rskhoshnood@yahoo.com; Department of Chemistry, Faculty of Science, Ferdowsi University of Mashhad Mashhad Iran pourayoubi@um.ac.ir; Institute of Physics of the Czech Academy of Sciences Na Slovance 2, 182 21 Prague 8 Czech Republic; Department of Chemistry, Lobachevsky State University of Nizhni Novgorod Gagarin Avenue 23 Nizhny Novgorod Russian Federation

## Abstract

Transport of Ag(i), Cd(ii), Co(ii), Cu(ii), Ni(ii), Pb(ii) and Zn(ii) cations across a bulk liquid membrane (BLM) containing *N*,*N*′-dibenzyl-*N′′*-(2,2,2-trifluoroacetyl)-phosphoric triamide (PTC) as a new carrier is studied by atomic absorption spectrometry. The results show selective and efficient transport of the copper(ii) cation from aqueous solution in the presence of the other cations. Various factors are optimized in order to obtain maximum transport efficiency. The PTC ligand is characterized by single crystal X-ray diffraction analysis, IR, NMR (^19^F, ^31^P, ^1^H, ^13^C) and mass spectroscopy. The complex formation reaction between copper(ii) and PTC is studied by a conductometric method, which shows the 1 : 1 stoichiometry for ligand and copper(ii).

## Abstract 2

Selective transport of Cu(ii) cation in the presence of six other cations across a bulk liquid membrane containing a novel phosphoric triamide carrier is studied.

## Graphical abstract

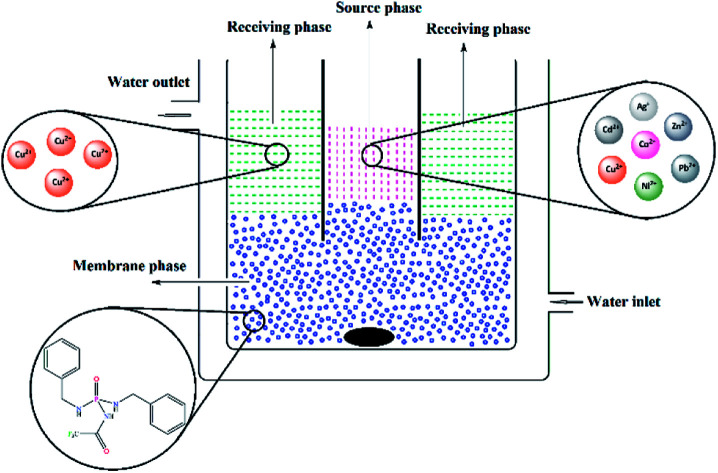

## Introduction

Copper is one of the essential micronutrients that participates in a wide range of metabolic processes. At least thirty Cu-containing enzymes are known, as redox catalysts (*e.g.* cytochrome oxidase, nitrate reductase) or dioxygen carriers. As copper is not biodegradable in natural conditions and accumulates in living organisms, its excess, as well as deficiency, can lead to diseases and biological disorders. Hence, its determination and elimination is very important for environmental protection.^1–4^ Towards this aim, various separation techniques are well-known, such as plasma atomic emission spectrometry,^5,6^ liquid chromatography,^7–9^ cloud point extraction,^10^ and ion transport methods using a bulk liquid membrane (BLM) system that has attracted attention in recent years.^11–15^ The use of a BLM containing specific metal ion carriers offers an alternative to the solvent extraction processes for the selective separation and concentration of metal ions from aqueous solutions.

Phosphoramides are studied due to their interesting structural and spectroscopic features,^16–18^ and owing to their extensive applications in coordination chemistry, catalysis and medicine.^19–22^ According to data from Cambridge Structural Database (CSD),^23^ thirty copper–phosphoric triamide complexes have been studied by X-ray diffraction analysis. Therefore, the potential use of a phosphoric triamide as a carrier in transport of copper can be expected through formation of related complex.

Often in transport processes, the transport rate of Cu(ii) ion reduces when Zn(ii) exists in the system. This inconvenience is minimized in the present work using a new synthetic phosphoric triamide carrier under optimized parameters of the transport process. In this competitive transport, a selective and highly efficient transmission of Cu(ii) cation is achieved, in the presence of Ag(i), Cd(ii), Co(ii), Ni(ii), Pb(ii) and especially Zn(ii). The synthesis and full characterization of the carrier compound, (CF_3_C(O)NH)(C_6_H_5_CH_2_NH)_2_P(O), are also investigated.

## Experimental

### Materials

The AgNO_3_ (99.8%), Co(NO_3_)_2_·6H_2_O (99.0%), Cu(NO_3_)_2_·3H_2_O (99.5%), Ni(NO_3_)_2_·6H_2_O (99.0%), Pb(NO_3_)_2_ (99.5%) nitrate salts were purchased from Merck, Zn(NO_3_)_2_·6H_2_O (98.0%) and Cd(NO_3_)_2_·4H_2_O (98.0%) were purchased from Aldrich. For preparation of PTC, phosphorus pentachloride (98%), trifluoroacetamide (97%), benzylamine (99%) and formic acid (97%) were used. Reagent grade chloroform (99.4%), dichloromethane (99.8%), 1,2-dichloroethane (99.8%) and nitrobenzene (99%) were tested as organic phases from Merck company; all chemicals were of the highest purity available and used without further purification. Doubly distilled deionized water was used throughout all experiments.

### Instruments and methods

The pH meter (Metrohm 691) with a glass electrode sensitive to the H_3_O^+^ ion was used, and calibrated with standard buffer solutions in pH = 4 and 7. Atomic absorption spectroscope (Hitachi Z-2000) with seven hallow cathode lamps (HCL) was used for analysis of metal cations concentrations. In all of the transport experiments, a water circulator was used around the cell, in order to control and fine adjust temperature. The samples were shaken on a shaker (Stuart CB-162). The conductance measurements were performed on a digital Metrohm conductivity apparatus (model 712) in a thermostated water-bath with a conductance temperature maintained within ±0.01 °C. The electrolytic conductance was measured using a cell consisting of two platinum electrodes to which an alternating potential was applied. A conductometric cell with a cell constant of 0.950 cm^−1^ was used throughout the studies. For X-ray diffraction experiment of PTC, a suitable single crystal was selected and mounted on a glass fiber. The experiment was carried out in a four-circle diffractometer Gemini of Oxford Diffraction, Ltd., equipped with a CCD detector Atlas S1, and using Cu Kα radiation (*λ* = 1.5418 Å) with a mirror monochromator. The crystal structure was solved using the charge flipping algorithm implemented in the program Superflip.^24^ Hydrogen atoms bonded to carbon atoms were kept in ideal positions, while the positions of hydrogen atoms attached to nitrogen atoms were refined using bond distance restrains of 0.87 Å. Anisotropic atomic displacement parameters were introduced for all non-hydrogen atoms, whereas for hydrogen atoms isotropic *U*_iso_ was evaluated as 1.2*U*_eq_ of the corresponding parent atom. Final models were refined with the program package JANA2006.^25^^1^H-, ^13^C-, ^19^F- and ^31^P-NMR spectra were recorded on a Bruker Avance DRX 400 spectrometer. Chemical shifts were determined relative to TMS for ^1^H and ^13^C and relative to CFCl_3_ for ^19^F, and 85% H_3_PO_4_ for ^31^P as the external standards. Infrared (IR) spectrum was recorded on a Buck 500 scientific spectrometer using a KBr disc. The mass spectrum was scanned on a Varian Mat CH-7 instrument at 70 eV.

### Synthesis of PTC

The (F_3_CC(O)NH)P(O)Cl_2_ reagent was prepared according to the procedure described in the literature, from the reaction between PCl_5_ and CF_3_C(O)NH_2_ in dry CCl_4_ in a reflux condition and then the treatment of HCOOH at ice bath temperature.^26,27^ The synthesis and melting point of (F_3_CC(O)NH)(C_6_H_5_CH_2_NH)_2_P(O) (PTC) was also reported^27^ and the procedure described here for PTC is similar to the literature method with a few modifications (in solvent and the reaction temperature). Moreover, we further study this compound with IR, NMR (^31^P, ^1^H, ^13^C, ^19^F) and single crystal X-ray diffraction. For the synthesis of PTC, to a solution of (F_3_CC(O)NH)P(O)Cl_2_ (2.06 mmol) in dry CH_3_CN, a solution of benzylamine (8.24 mmol) in the same solvent was added dropwise at 273 K. After stirring for 4 h, the solvent was evaporated in vacuum and the residue was washed with distilled water. Single crystals were obtained in CH_3_OH/CH_3_CN (4 : 1 v/v) after slow evaporation at room temperature. Mp 180 °C.

IR (KBr, cm^−1^): 3271, 2945, 2346, 1729, 1485, 1384, 1307, 1193, 957, 808. ^31^P{^1^H}-NMR (162 MHz, DMSO-d_6_, ppm): 6.61 (s). ^1^H-NMR (400 MHz, DMSO-d_6_, ppm): 4.03 (dd, *J* = 12.4, 7.1 Hz, CH_2_, 4H), 5.34 (dt, *J* = 13.3, 7.2 Hz, NH, 2H), 7.15–7.37 (m, CH, 10H), 10.34 (s, NH, 1H). ^13^C-NMR (101 MHz, DMSO-d_6_, ppm): 43.74 (s, CH_2_), 115.30 (qd, *J* = 289.6, 12.3 Hz, CF_3_), 126.73 (s), 127.28 (s), 128.12 (s), 140.72 (d, *J* = 5.4 Hz), 157.33 (qd, *J* = 38.4, 1.3 Hz, CO). ^19^F-NMR (376 MHz, DMSO-d_6_, ppm): −74.54. MS (70 eV): *m*/*z* (%) = 371 (33) [M]^+^, 370 (26) [M − 1]^+^, 369 (50) [M − 2]^+^, 276 (40) [M −  CF_3_CN]^+^, 105 (100) [C_7_H_7_N]^+^, 77 (73) [C_6_H_5_]^+^. CCDC number: 1880340.

### Construction of BLM

**Fig. 1:**
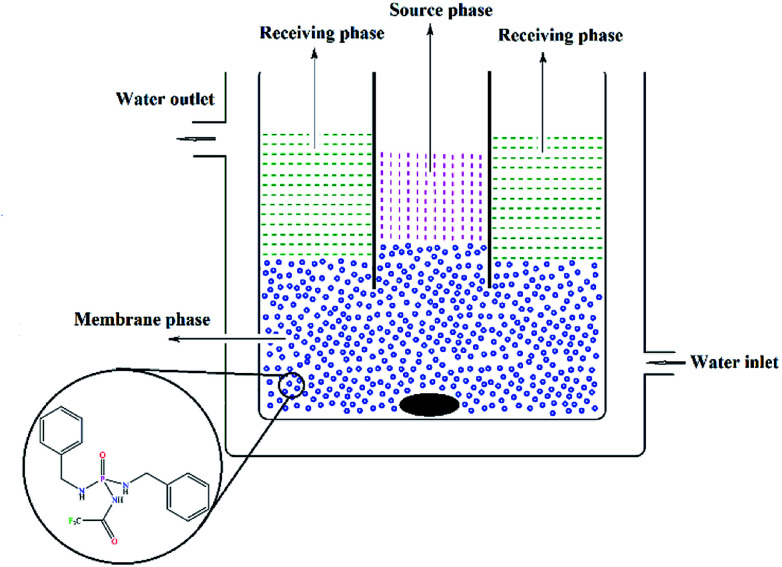
The concentric cell (BLM) for transport experiments (a relatively thick layer of immiscible fluid is used to separate the source and receiving phases).

The transport experiment was carried out by a double-standard concentric cell in which the source aqueous phase (10 ml) and the receiving aqueous phase (30 ml) were separated by an organic membrane phase (50 ml). The experimental set-up^28,29^ was a double jacket cylindrical glass cell (4.5 cm diameter) holding a glass tube (with a diameter of 2.25 cm) for separating the two aqueous phases. In a BLM, a relatively thick layer of immiscible fluid is used to separate the source and receiving phase. Actually, there is no means of support for the membrane phase and it is kept apart from the external phases only by means of its immiscibility. The glass concentric cell was enclosed with a water jacket, at the temperature setting in 25 °C. The details of the cell (BLM) are shown in Fig. 1.

The source aqueous phase consisted of a buffer solution at pH = 4.9 (acetic acid/sodium acetate) containing an equimolar mixture of seven metal cations, each at the concentration of 1 × 10^−2^ mol L^−1^. The receiving phase consisted of a buffer solution at pH = 3.0 (formic acid/sodium hydroxide). The membrane phase contained the PTC carrier (1 × 10^−3^ mol L^−1^) in organic solvent (CHCl_3_, DCM, 1,2-DCE, NB). In each experiment, the organic phase was stirred for 24 h at 60 rpm.

Samples of source and receiving phases were analyzed by atomic absorption spectroscopy after each transport run. A series of standard solutions, which were made similarly, were also analyzed by atomic absorption spectroscopy. With analysis of cations in the source and receiving phases, the amounts of the metal cations within the membrane phase were measured. According to the measured values of the metal cations in liquid organic membrane, source aqueous phase and recipient aqueous phase, the speed and percentage of transport for the cations were calculated. The reported results are the averages from three experiments.

### Conductometric method

The experimental procedure to determine the stability constants of complexes has been done according to a previously reported method.^30^ Briefly as follows: to a solution of metal salt (1 × 10^−4^ M) in a titration cell, the PTC solution (2 × 10^−3^ M) has been added. Then the solution was carried out to the titration cell by using a microburette, rapidly. The conductances of the solutions were measured initially and after each transfer in the desired temperature.

## Results and discussion

### X-ray crystal structure description

**Scheme 1:**
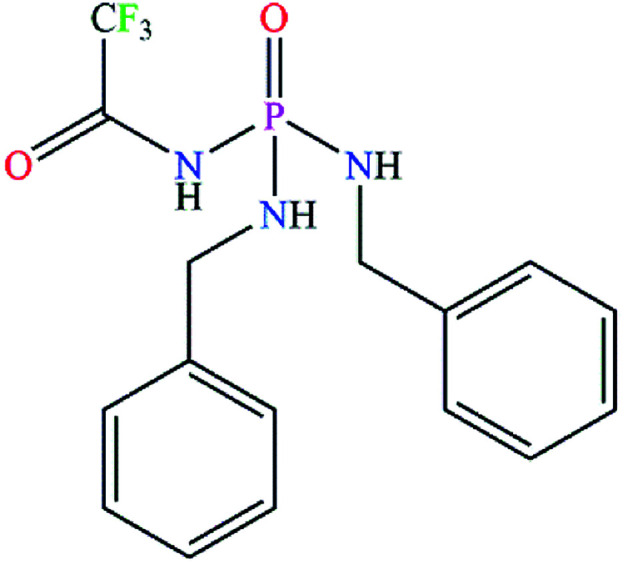
The chemical structure of PTC.

**Fig. 2:**
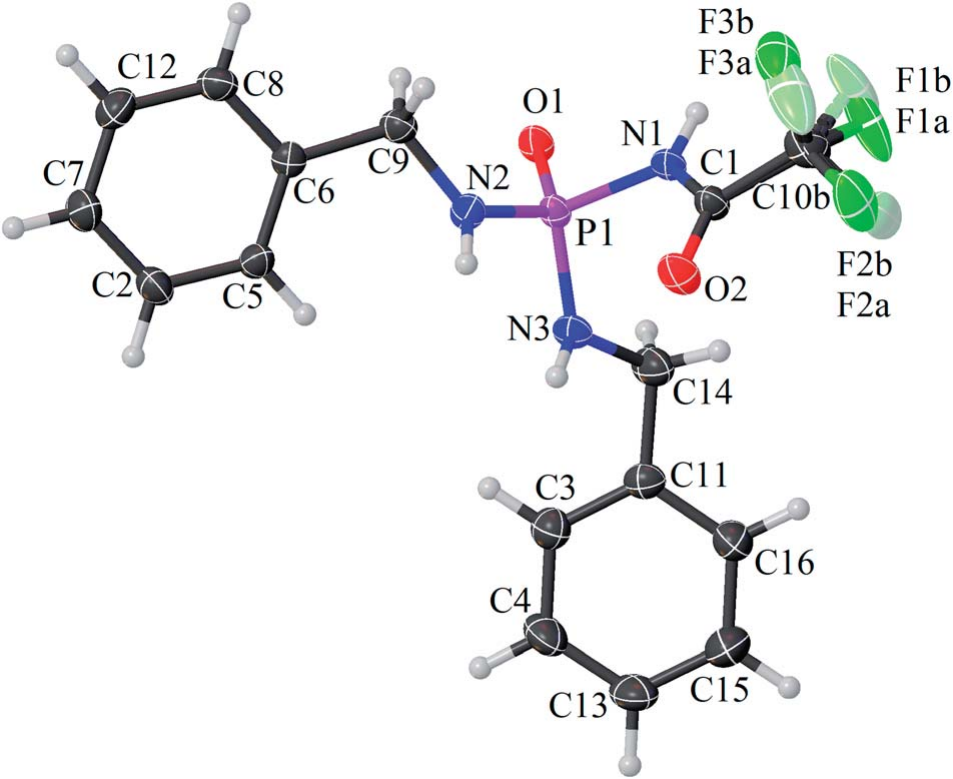
Displacement ellipsoid plot (50% probability) and atom-numbering scheme for PTC. The H atoms are drawn as small spheres of arbitrary radii. Selected bond lengths (Å) are P1–O1 = 1.4718(16), P1–N1 = 1.7306(19), P1–N2 = 1.616(2) and P1–N3 = 1.615(2), and selected bond angles (°) are O1–P1–N1 = 103.29(9), O1–P1–N2 = 116.39(9), O1–P1–N3 = 116.53(10), N1–P1–N2 = 109.35(10), N1–P1–N3 = 107.28(10) and N2–P1–N3 = 103.66(10).

The achiral (CF_3_C(O)NH)(C_6_H_5_CH_2_NH)_2_P(O) phosphoric triamide (with chemical structure as shown in Scheme 1) crystallizes in the chiral space group *P*2_1_2_1_2_1_, with *Z* = 4. Crystallographic data and structure refinement parameters are listed in Table 1. Fig. 2 shows a thermal ellipsoid plot of one complete molecule, which is present in the asymmetric unit, and selected bond lengths and angles are given in the caption of the figure.

**Table 1:** Crystallographic data for PTC

Formula weight	371.3
Temperature	119.9(6) K
Empirical formula	C_16_H_17_F_3_N_3_O_2_P
Wavelength	1.5418 Å
Crystal system	Orthorhombic
Space group	*P*2_1_2_1_2_1_
Unit cell dimensions	*a* = 4.96980(10) Å
	*b* = 9.7925(3) Å
	*c* = 34.4713(9) Å
	*α* = *β* = *λ* = 90.00°
Volume	1677.61(8) Å^3^
*Z*	4
Density (calculated)	1.4696 g cm^−3^
Absorption coefficient	1.896 mm^−1^
*F*(000)	768
Crystal size	0.258 × 0.046 × 0.042 mm^3^
Crystal color, habit	Colorless, block
Theta range for data collection	4.69 to 67.11°
Index ranges	−5 ≤ *h* ≤ 5, −11 ≤ *k* ≤ 11, −41 ≤ *l* ≤ 41
Reflections collected	20 216
Independent reflections	2953 [*R*(int) = 0.0522]
Completeness to theta = 67.1°	99.8%
Absorption correction	Semi-empirical from equivalents
Max. and min. transmission	1 and 0.868
Data/restraints/parameters	2953/3/235
Goodness-of-fit on *F*^2^	1.28
Final *R* indices [*I* > 3*σ*(*I*)]	*R*_1_ = 0.0323, *wR*_2_ = 0.0783
R indices (all data)	*R*_1_ = 0.0382, *wR*_2_ = 0.0815
Largest diff. peak and hole	0.19 and −0.22 e Å^−3^

The P=O bond length is standard for phosphoramide compounds,^31^ and the P–N bond of the C(O)NHP(O) segment (P1–N1) is longer than the two other P–N bonds (P1–N2 and P1–N3), as was observed in (C(O)NH)(N)_2_P(O)-based compounds.^17^

In the C(O)NHP(O) segment, the NH unit adopts a *syn* orientation with respect to the P=O group, while the two other NH units in the molecule show an *anti* orientation relative to P=O. The phosphorus atom has a distorted tetrahedral (N)_3_P(O) environment with the bond angles at the P atom in the range of 103.29(9)° (O1–P1–N1) to 116.53(10)° (O1–P1–N3). The nitrogen atoms show practically planar environments, reflected in the bond-angle sum close to 360°. The CF_3_ group exhibits disorder. The rigid body refinement of two distinct CF_3_ positions yields occupancy 0.772 and 0.228 for the major and minor occupied parts, respectively.

**Fig. 3:**
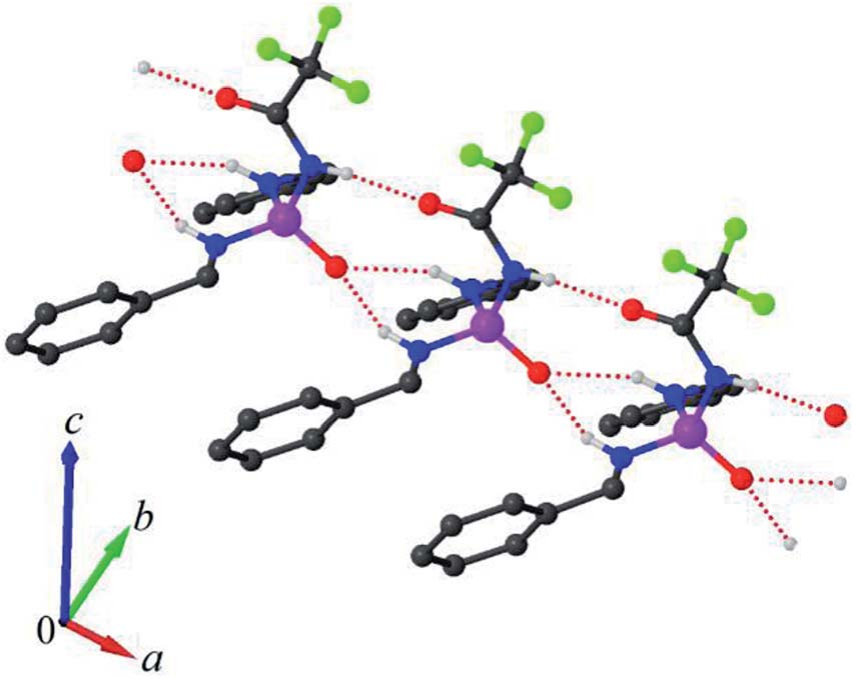
A view of one-dimensional array of molecules, built from NH⋯O hydrogen bonds. The weakly occupied atoms and hydrogen atoms not involved in hydrogen bonding were omitted for the sake of clarity.

In the crystal packing, adjacent molecules are linked *via* ((C_6_H_5_CH_2_)N–H)_2_⋯O=P and CF_3_C(O)⋯HNC(O)CF_3_ hydrogen bonds, forming a linear arrangement along the *a* axis (Fig. 3). This arrangement includes non-centrosymmetric R_2_^1^(6) and R_2_^2^(10) graph-set motifs, together with a C4 chain motif. The hydrogen-bond parameters are listed in Table 2.

**Table 2:** Hydrogen-bond geometry (Å, °)^a^

D—H⋯A	D—H	H⋯A	D⋯A	D—H⋯A
N1— H1n1⋯O2^i^	0.870(11)	1.978(9)	2.839(3)	170(2)
N2—H1n2⋯O1^ii^	0.870(7)	2.097(15)	2.903(2)	154(2)
N3—H1n3⋯O1^ii^	0.870(4)	2.244(16)	3.031(2)	150(2)

a Symmetry codes: (i) *x* + 1, *y*, *z*; (ii) *x* − 1, *y*, *z*.

### NMR study

The ^31^P and ^19^F signals appear at 6.61 and −74.54 ppm, respectively that confirm the chemical structure and purity. The chemical shifts are comparable with the phosphorus signal at 9.68 ppm for (C_6_H_5_NH)(C_6_H_5_CH_2_NH)_2_P(O)^32^ and fluorine signal at −74.67 ppm for (CF_3_C(O)NH)P(O)(NH)_2_C_3_H_4_(CH_3_)_2_.^33^

**Fig. 4:**
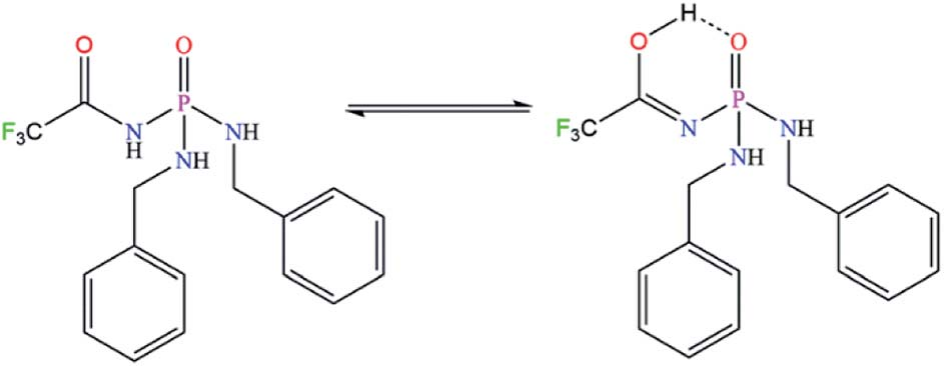
The possible tautomeric equilibrium in solution.

The NH proton of the C(O)NHP(O) segment is revealed as a broad signal centred at the high frequency of 10.34 ppm, due to its mobility caused by the electronegative CF_3_ group, which leads to its acidic character, and also due to possibility of the tautomeric equilibrium in solution (as illustrated in Fig. 4).

The NH protons of the C_6_H_5_CH_2_NH groups appear at 5.34 ppm as a doublet of triplets fine structure due to H–H and P–H couplings.

In the ^13^C NMR spectrum, the signals of CF_3_ (at 115.30 ppm) and CO (at 157.33 ppm) both appear as a qd pattern, due to the couplings with fluorine and phosphorus nuclei. The *ipso*-carbon atom of benzyl group at 140.72 ppm shows a three-bond separation phosphorus–carbon coupling constant (^3^*J*_P–C_ = 5.4 Hz).

### Transport properties

Although the effect of various carriers on the transport of alkali and alkaline earth metal cations through liquid membranes has been reported so far, relatively few carriers offer a selective and efficient transport of transition or heavy metal ions,^34,35^ however they are also important from biological, medicinal, environmental and industrial points of view.^36,37^

Competitive transport of seven metal cations among four membrane phases of chloroform (CHCl_3_), dichloromethane (DCM), 1,2-dichloroethane (1,2-DCE) and nitrobenzene (NB) was evaluated in the presence of the PTC carrier. A pH gradient was utilized to facilitate transport of the metal ions across the membrane that controls the return penetration of protons from the receiver aqueous phase (with pH = 3) to the source aqueous phase (pH = 4.9).

**Fig. 5:**
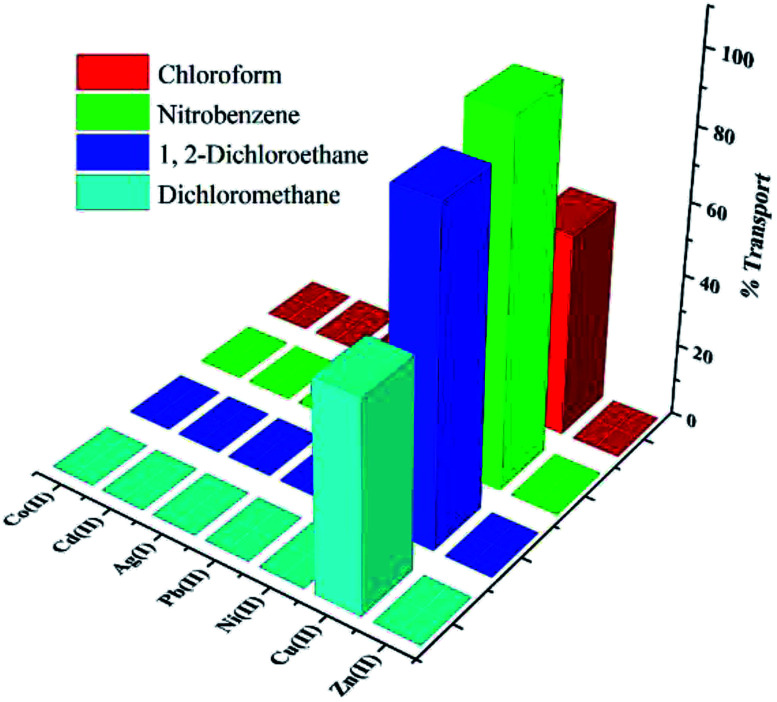
Transport percentages for seven metal cations by PTC carrier in different organic solvents.

In the source–organic interface, the metal ion comes in contact with the protonated carrier, and forms a complex with deprotonated carrier. The complex penetrates from organic phase to the more acidic receiver phase, and the metal ions are replaced by protons. Then, protonated carrier returns to the organic phase and the cycle is repeated, leading to an increase in the concentration of transferred metal ions in the receiver phase. In fact, the transfer occurs from the source phase to the membrane and then from the membrane phase to the recipient phase. From the obtained results, the transfer mechanism of Cu(ii) ion is suggested as the pH gradient driven. The amounts of ions in receiving and membrane phases and the rate of transport for seven mentioned metal cations in different organic membrane solvents (CHCl_3_, DCM, 1,2-DCE and NB) are listed in Table 3 and visualized in Fig. 5. Transports of Ag(i), Cd(ii), Co(ii), Cu(ii), Ni(ii), Pb(ii) and Zn(ii) cations in the absence of PTC (*e.g.* in CHCl_3_, DCM, 1,2-DCE and NB neat solvents) were studied by atomic absorption spectrometry. The results show that none of the cations is transferred to the received phase.

**Table 3:** The results of competitive metal-ion transport of the seven-metal cations across different bulk liquid membranes using (CF_3_C(O)NH)(C_6_H_5_CH_2_NH)_2_P(O) carrier

Solvents	Cations						
	Co(ii)	Cd(ii)	Ag(i)	Pb(ii)	Ni(ii)	Cu(ii)	Zn(ii)
**Chloroform**							
Receiving, %^a^	—b	—b	0.05	0.02	—b	55.92	—b
Membrane, %^c^	45.03	50.88	42.34	49.61	0.32	44.07	55.70
*J* d/(mol per h)	—b	—b	0.06	0.02	—b	7.00	—b

**Dichloromethane**							
Receiving, %^a^	—b	—b	0.05	0.02	—b	58.11	—b
Membrane, %^c^	43.52	49.34	38.80	47.79	0.14	41.88	54.53
*J* d/(mol per h)	—b	—b	0.06	0.02	—b	7.30	—b

**1,2-Dichloroethane**							
Receiving, %^a^	—b	—b	0.05	—b	—b	91.31	—b
Membrane, %^c^	9.23	70.84	31.72	15.69	0.16	8.69	29.48
*J* d/(mol per h)	—b	—b	0.06	—b	—b	11.40	—b

**Nitrobenzene**							
Receiving, %^a^	—b	—b	0.05	—b	—b	99.69	—b
Membrane, %^c^	43.52	21.94	22.73	1.66	0.05	0.03	19.59
*J* d/(mol per h)	—b	—b	0.06	—b	—b	12.50	—b

a Percent of metal cation in the receiving phase after 24 h.

b The hyphenated symbols mean that values about zero.

c Percent of metal cation in the membrane phase after 24 h.

d All *J* (flux rate) values are ×10^−7^.

### Effect of solvent

The solvent used in the membrane phase plays an important role in conveying and separating metal ions.^38,39^ The solvent should have high distribution coefficient, should be immiscible with the aqueous phase, with low viscosity and volatility.^40^ Thus, various parameters of a solvent in the organic liquid membrane influence on the transport properties. Also, mobility coefficient of species in boundary layers and value of complex formation constant (*K*_f_) between the cation and ligand in organic membrane phase^41^ are effective on the transport. The boundary layer composition, and distribution of cations in the organic solvent, is affected by dielectric constant and polarity of the solvent.^42^ In solvents with higher polarity, the rate of ion transfer is increased because of the higher ability to dissolve the complex formed between the carrier molecule and cation.

For four solvents tested in this work (Table 3), it was found that the optimal solvent is NB, where the transport of Cu(ii) ion from the aqueous source phase into the receiving phase after 24 h is 99.69%, while other competing ions are transported to a very small extent.

The sequence of transport rates for Cu(ii) cation in organic solvents is as NB > 1,2-DCE > DCM > CHCl_3_, in agreement with the polarity and dipole moments: *μ* = 4.02 and *E*^N^_T_ = 0.356 for NB, *μ* = 1.86, *E*^N^_T_ = 0.327 for 1,2-DCE, *μ* = 1.55, *E*^N^_T_ = 0.309 for DCM, and *μ* = 1.54, *E*^N^_T_ = 0.259 for CHCl_3_. Also, because the studied PTC is a polar compound, it should be better dissolved in solvents with larger polarization. Moreover, due to higher dielectric constant of NB (*ε* = 34.82) compared with 1,2-DCE (*ε* = 10.36), DCM (*ε* = 8.93) and CHCl_3_ (*ε* = 4.81), the possibility of ion-pair forming in NB solvent is lower and consequently transmission rate of Cu(ii) ions increases. The selectivities of the organic liquid membranes for the metal cations studied are compared in Table 4. With all solvents, the selectivity of PTC for Cu(ii) cation is superior to that for the other cations.

**Table 4:** Selectivity sequences for competitive transport of metal cations across bulk liquid membranes

Solvents	Selectivity
CHCl_3_	Cu(ii) > Ag(i) > Pb(ii)
NB	Cu(ii) > Ag(i)
1,2-DCE	Cu(ii) > Ag(i)
DCM	Cu(ii) > Ag(i) > Pb(ii)

### The study of complex formation between PTC and Cu^2+^

**Fig. 6:**
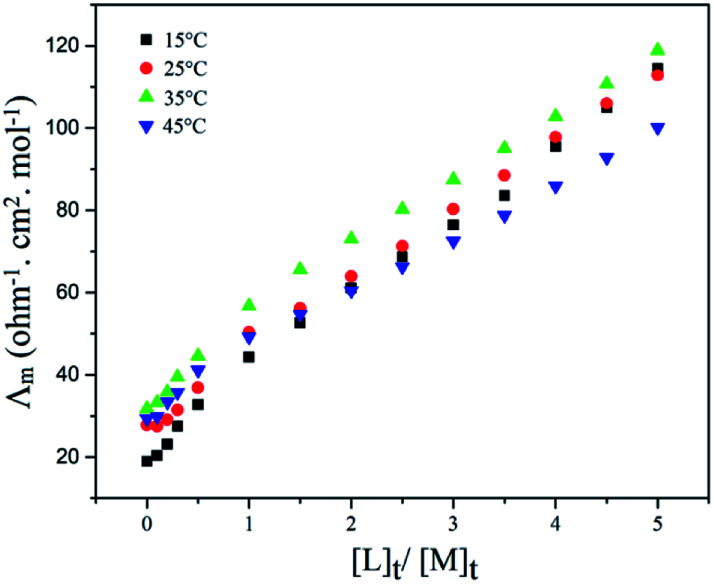
Molar conductance–mole ratio plots for the PTC–Cu(ii) complex in NB at different temperatures.

The complex formation between PTC and Cu(ii) ion was investigated by conductometry experiments in NB at different temperatures, with measuring the molar conductivity (*Λ*_m_) as a function of ligand to cation molar ratio ([L]_t_/[M]_t_), Fig. 6. As seen in Fig. 6, with addition of PTC to cation solution, the molar conductivity increases which indicates that the PTC–Cu(ii) complex is more mobile than free solvated Cu^2+^ cation. Moreover, the breaking of the curves is nearly to 1 and the stoichiometry ratio about 1 : 1 was observed for the complex formed.

The stability constants of the PTC–Cu complex were calculated using GENPLOT program,^43^ which yield log *K*_f_ of 2.81 ± 0.17, 2.82 ± 0.14, 2.93 ± 0.10 and 2.58 ± 0.24 in 15, 25, 35 and 45 °C, respectively. The log *K*_f_ value indicates that PTC is a suitable carrier to separate Cu(ii) cation in a bulk liquid membrane system. The details of calculation of the stability constants of complexes by the conductometric method have been described in pervious report.^44^

## Conclusions

The competitive transport of Ag(i), Cd(ii), Co(ii), Cu(ii), Ni(ii), Pb(ii) and Zn(ii) metal cations through the various bulk liquid membranes containing (CF_3_C(O)NH)(C_6_H_5_CH_2_NH)_2_P(O) (PTC) as a new carrier was studied and this ligand was found to be very suitable carrier for competitive transport of Cu^2+^ metal cation. Also, this study demonstrates the usefulness of the liquid membrane technique for combining extraction and stripping operations in a single process. The results of the simultaneous transport show that the rate and selectivity of the ion transport are strongly influenced by the nature of the membrane solvent. The selectivity of the membrane systems for Cu^2+^ cation in presence of ion mixture depends on the solvents as follows: NB > 1,2-DCE > DCM > CHCl_3_. The percentage of copper transport reached 99.69% in optimum membrane solvent of NB at temperature of 298 K after 24 h.

## Conflicts of interest

The authors have no conflicts of interest regarding this work.

## Supplementary Material

RA-009-C8RA09118H-s001
